# The current and future cancer burden in the Gulf Cooperation Council (GCC) countries

**DOI:** 10.1002/cam4.70141

**Published:** 2024-09-16

**Authors:** Saleh A. Alessy, Saleh A. Alqahtani, Jerome Vignat, Amid Abuhmaidan, Amani E. L. Basmi, Najla Al Lawati, Ameera Ali A‐Nooh, Wael Shelpai, Samar Alhomoud, Ali Al‐Zahrani, Freddie Bray, Ariana Znaor

**Affiliations:** ^1^ Public Health Department, College of Health Sciences Saudi Electronic University Riyadh Saudi Arabia; ^2^ Centre for Cancer, Society & Public Health, Faculty of Life Sciences and Medicine King's College London UK; ^3^ Division of Research & Innovation King Faisal Specialist Hospital and Research Center Riyadh Saudi Arabia; ^4^ Organ Transplant Center of Excellence King Faisal Specialist Hospital and Research Center Riyadh Saudi Arabia; ^5^ Division of Gastroenterology and Hepatology Weill Cornell Medicine New York USA; ^6^ Cancer Surveillance Branch The International Agency for Research on Cancer Lyon France; ^7^ Qatar National Cancer Registry Doha Qatar; ^8^ Kuwait National Cancer Registry Kuwait City Kuwait; ^9^ Oman National Cancer Registry Muscat Oman; ^10^ Bahrain Cancer Registry Manama Bahrain; ^11^ National Cancer Registry Dubai United Arab Emirates; ^12^ Section of Colorectal Surgery King Faisal Specialist Hospital and Research Center Riyadh Saudi Arabia

**Keywords:** cancer, epidemiology, Gulf Cooperation Council, incidence, mortality, pattern

## Abstract

**Background:**

Cancer is a leading cause of morbidity and mortality in the Gulf Cooperation Council (GCC) countries. This study aims to provide cancer incidence and mortality estimates in 2020 in the GCC countries alongside future projections for 2040 to shape cancer control policy in the region.

**Methods:**

The estimated numbers of new cancer cases and deaths were extracted from the GLOBOCAN database developed by the International Agency for Research on Cancer; new cancer cases, cancer deaths, and corresponding age‐standardized incidence and mortality rates for the year 2020 are presented.

**Results:**

An estimated 42,475 new cancer cases and 19,895 deaths occurred in the GCC countries in 2020, with corresponding age‐standardized incidence and mortality rates of 96.5 and 52.3 per 100,000, respectively. Female breast (16%), colorectal (13%), and thyroid (9%) were the most common types of cancer in the GCC countries, accounting for almost 40% of all cancer incidence. Colorectal (14%) followed by breast cancer (9%) were the leading causes of cancer death, though the magnitude of rates of the major cancer types varied substantially across the GCC countries. Even if we assume rates in the region will remain unchanged over the next two decades, the cancer burden in the GCC will increase by 116% (Saudi Arabia) to 270% (Qatar), reaching nearly 104,000 cancer cases by the year 2040.

**Conclusion:**

The sharp increase in the estimated cancer incidence and mortality predicted over the next decades in the region requires workforce and financial planning for the healthcare systems in the constituent countries, alongside broader strengthening of national cancer prevention and control efforts.

## 1 INTRODUCTION

The Gulf Cooperation Council (GCC) countries are located in the Arabian Peninsula and consist of six countries (Saudi Arabia, Qatar, the United Arab Emirates (UAE), Bahrain, Oman, and Kuwait), all combined are home to a population of over 55 million.^1^ All GCC countries have a very high human development index, with the healthcare systems in these countries witnessing rapid reforms that have resulted in better control of communicable and non‐communicable diseases and, subsequently, longer life expectancies.^2^ Cancer services have, in parallel, expanded with investments in free universal healthcare coverage and the development of national strategies for the prevention, early detection, and management of cancer.^3^ Despite such progress, cancer is a leading cause of premature morbidity and mortality in the GCC countries. With populations both aging and increasingly adopting unhealthy lifestyles, cancer has become a major threat to public health and economic growth in the region.^4^, ^5^, ^6^, ^7^

Assessing the specific‐context cancer burden assessment and projecting future trends are crucial for data‐driven cancer control policies and resource allocation. There is, however, limited research comparing cancer incidence and mortality patterns between the GCC countries.^8^, ^9^ Several region studies have reported marked national variations in the scale and profile of cancer between GCC countries,^8^ despite the underlying populations sharing similar cultures and healthcare systems.

The GLOBOCAN dataset is developed, hosted, quality‐assured, and publicly made available by the International Agency for Research on Cancer (IARC), enabling researchers to compare and monitor cancer burden globally and across many countries.^10^, ^11^ With comprehensive descriptions needed to strengthen research initiatives and cancer control policies, this study examines national cancer incidence and mortality patterns in the six GCC countries using the most recent GLOBOCAN estimates for 2020 developed by the IARC,^11^ alongside future cancer projections until 2040.

## 2 METHODOLOGY

The number of new cancer cases and deaths were extracted from IARC's GLOBOCAN 2020 database for the six GCC countries (Saudi Arabia, Qatar, UAE, Bahrain, Oman, and Kuwait) by the most common cancer type (defined according to the list of the cancer sites available in the GLOBOCAN 2020 database with corresponding International Classification of Disease, ICD‐10 codes) and all cancer sites combined, sex and 18 age groups (0–4, 5–9, …, 80–84, 85 and over), with corresponding population data based on projections provided by the United Nations (UN).^12^ The quality of the national estimates depends on the coverage, accuracy, and timeliness of the recorded incidence and mortality data in each country. In terms of incidence, all GCC countries have national cancer registration systems, with variations in data collection and software usage to process registry data.^6^, ^13^ The GLOBOCAN mortality estimates for all GCC countries, as presented here, were necessarily derived based on corresponding cancer incidence estimates.^13^

To allow comparisons between countries adjusted for differences in age structure, age‐standardized incidence and mortality rates (ASRs) per 100,000 person‐years were calculated using the Segi–Doll world standard population.^11^ To reduce random fluctuations in the mortality trends, the annual ASRs were smoothed using LOESS regression by country, sex, and cancer site. Lastly, we present predictions of all‐cancer incidence and mortality for the year 2040 by applying country‐, sex‐ and age‐specific incidence rates to the demographic projections for the population provided by the UN up to the year 2040,^14^, ^15^ assuming different scenarios (rates in 2020 will remain constant over the following two decades; 1%–3% annual increases and 1%–3% annual decreases in rates). These data sources and the hierarchy of methods used to compile the cancer estimates in the GLOBOCAN 2020 database have been described elsewhere.^11^

The results are presented by each GCC country and aggregated for the region. The Global Cancer Observatory (https://gco.iarc.fr) includes facilities for the tabulation and graphical visualization of the GLOBOCAN database, including explorations of the current and future burden for 36 cancer types and all cancers combined, including non‐melanoma skin cancer (ICD‐10 C44 excluding basal‐cell carcinomas).

## 3 RESULTS

### 3.1 Overall burden and key cancers in the GCC

**FIGURE 1:**
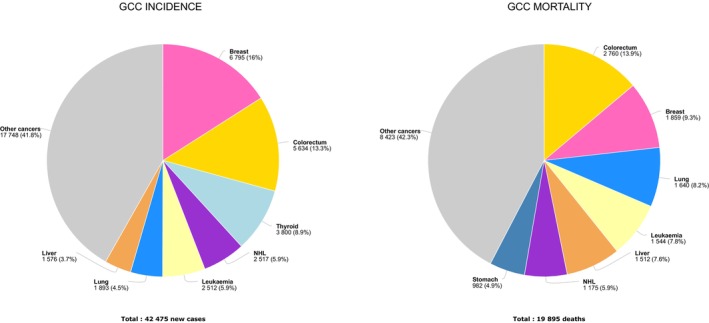
Proportion of cancer incidence and mortality in the GCC countries, GLOBOCAN 2020.

**FIGURE 2:**
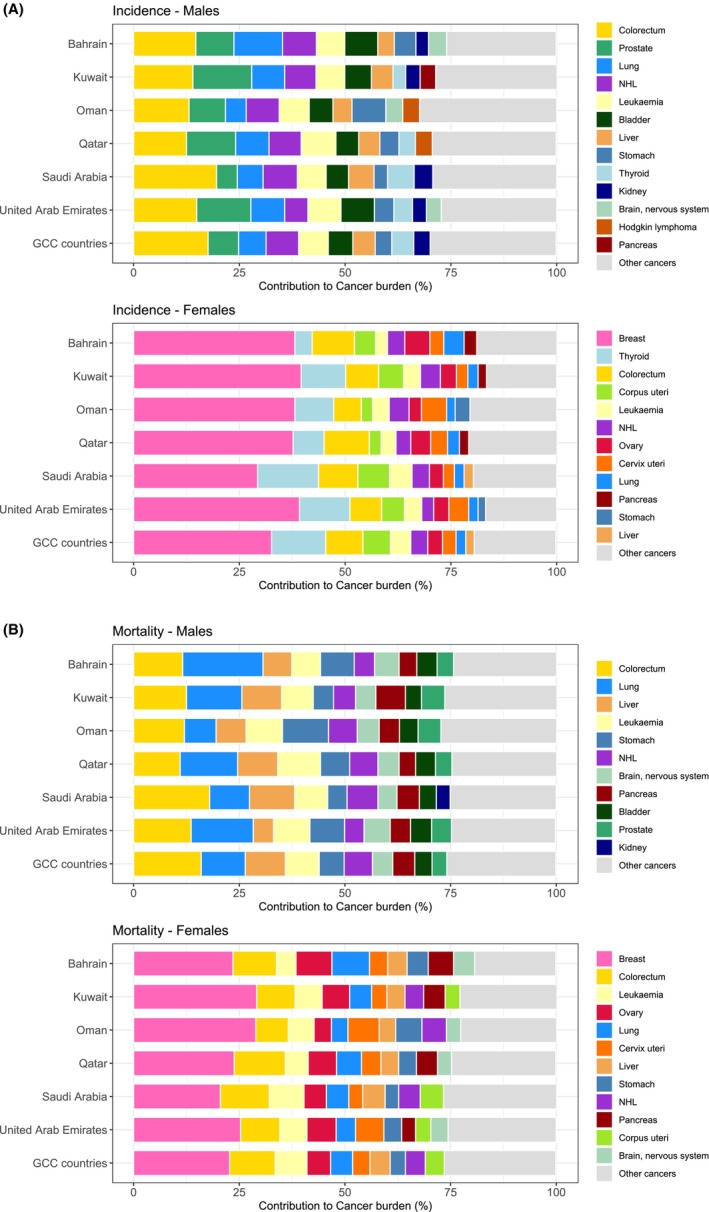
(A) The most common cancers (incidence) in each GCC country and combined, GLOBOCAN 2020. (B) Proportions of the most common cancers (mortality) in each of the GCC countries and combined, GLOBOCAN 2020.

There were 42,475 new cancer cases in the GCC countries in 2020, with almost two‐thirds occurring in Saudi Arabia, the most populated country in GCC (Table 1); the corresponding ASR for cancer incidence was 96.5 per 100,000 (both sexes). There were an estimated 19,895 deaths in the GCC countries in 2020, with 65% occurring in Saudi Arabia; the mortality rate (both sexes) was 52.3 per 100,000. Cancers of the breast (16%), colorectum (13%), and thyroid (9%) were the most frequent types of cancer incidence in the GCC countries, accounting for almost 40% of the overall burden (Figure 1, Tables S1–S8). The hematological cancers—non‐Hodgkin lymphoma (NHL) and leukemia—were the fourth and fifth most common cancers in the region, respectively. Colorectal cancer was the most common cancer among males in the region, with breast cancer the most frequent in females (Figure 2A). Thyroid cancer was the second and colorectal, the third most common cancer among females in all countries except Qatar, where the order was inversed, and in Bahrain, where thyroid cancer was the sixth most common. In men, prostate and lung cancers were either the second or the third most common cancers in UAE, Kuwait, Qatar, and Bahrain (Figure 2A). NHL and leukemia, respectively, were the second and third most common sites in men in Saudi Arabia, while gastric cancer was the third most common cancer among men in Oman (Figure 2A, Tables S6 and S7).

**TABLE 1:** Cancer incidence and mortality in the Gulf Cooperation Council countries in GLOBOCAN 2020, excluding non‐melanoma skin cancer.

Country	Population size	Incidence						Mortality					
		Male		Female		Both sexes		Male		Female		Both sexes	
		Cases	ASR	Cases	ASR	Cases	ASR	Cases	ASR	Cases	ASR	Cases	ASR
Bahrain	1,701,583	564	107.4	641	130.4	1205	111.5	311	67.8	281	65.6	592	64.2
Kuwait	4,270,563	1829	100.8	1995	144.1	3824	115.1	947	59.8	771	71	1718	63.5
Oman	5,106,622	2198	106.6	1466	107.3	3664	101.2	1344	72.5	675	56.5	2019	64.1
Qatar	2,881,060	894	100.9	578	138.3	1472	106.6	464	61	239	76.9	703	64.4
Saudi Arabia	34,813,867	14,066	87.8	13,512	110.8	27,578	95.2	7635	53	5351	49.7	12,986	50.9
UAE	9,890,400	2105	80.7	2627	168.9	4732	104.8	998	46.2	879	78.2	1877	55.1
All GCC countries	58,664,095	21,656	88	20,819	117.7	42,475	96.5	11,699	53.2	8196	53.8	19,895	52.3

Abbreviations: ASR, age‐standardized rates per (world standard population) per 100,000; M:I, mortality‐to‐incidence; UAE, United Arab Emirates.

Cancers of the colorectum (14%), breast (9%), and lung (8%) were the leading causes of cancer death in the GCC region for both sexes combined (Figure 1). In terms of cancer mortality among men, colorectal cancer was the leading cause of death in Saudi Arabia and Oman, while lung cancer was the leading cause of death in UAE, Kuwait, Qatar, and Bahrain (Figure 2B). In females, breast cancer was the leading cause of cancer death across all GCC countries (Figure 2B).

### 3.2 Variations in cancer incidence and mortality rates

**FIGURE 3:**
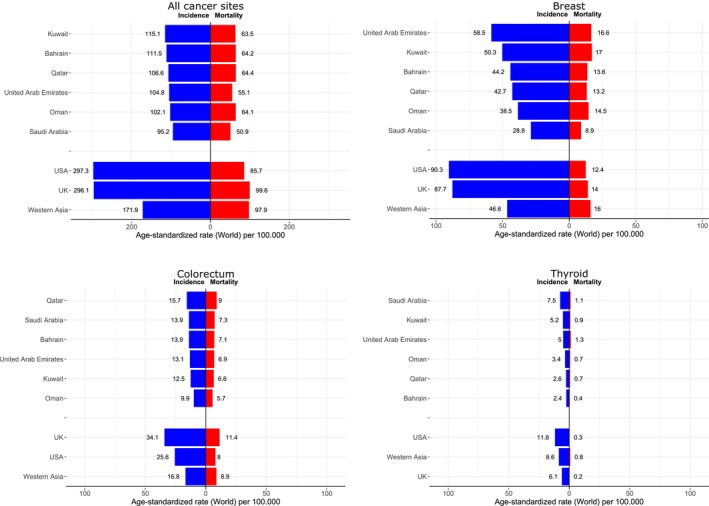
ASR for incidence and mortality for all cancer sites, breast, colorectum, and thyroid cancers in the GCC countries compared to other countries and regions, GLOBOCAN 2020.

As seen in Figure 3 and Table 1, overall incidence rates in GCC countries were very low compared to the United States (US) and the United Kingdom (UK), although the ASRs varied between countries, with the highest rates (in both sexes) seen in Kuwait (115.1), followed by Bahrain (111.5), and the lowest in Saudi Arabia (95.2). Mortality rates were also considerably lower than the US and the UK, with the highest rates (in both sexes) in Qatar (64.4)—similar to those seen in Bahrain, Oman, and Kuwait—with the lowest in Saudi Arabia (50.9). Breast cancer incidence rates were highest in the UAE (58.5), followed by Kuwait (50.3), and lowest in Saudi Arabia (28.8). The rates were considerably lower than those in the US (90.3), and the UK (87.7) but higher than in western Asia (46.6) (Figure 3, Table S1). In contrast, there were similar or higher breast cancer mortality rates in most GCC countries (e.g., Kuwait: 17.0; UAE: 16.6) relative to the US and the UK (Figure 3, Table S1). A similar pattern was seen for colorectal cancer, with incidence rates in the GCC countries lower than their US and UK counterparts, whereas mortality rates were similar or higher in some GCC countries. The highest colorectal incidence rate was in Qatar (15.7), followed by Saudi Arabia (13.9), with rates lowest in Oman (9.9); these rates were, however, still lower than those seen in the US (25.6), the UK (34.1) and western Asia (16.8) (Figure 3). Thyroid cancer rates in the GCC countries (overall) was 6.1/100,000, with the highest thyroid incidence ASR observed in Saudi Arabia (7.5), followed by Kuwait (5.2) and the lowest ASR seen in Bahrain (2.4) (Figure 3, Table S5). The mortality rates from thyroid cancer in GCC were higher in some GCC countries than in the US or the UK, with variations between GCC countries (Figure 3).

### 3.3 Variations in cancer incidence by sex

**FIGURE 4:**
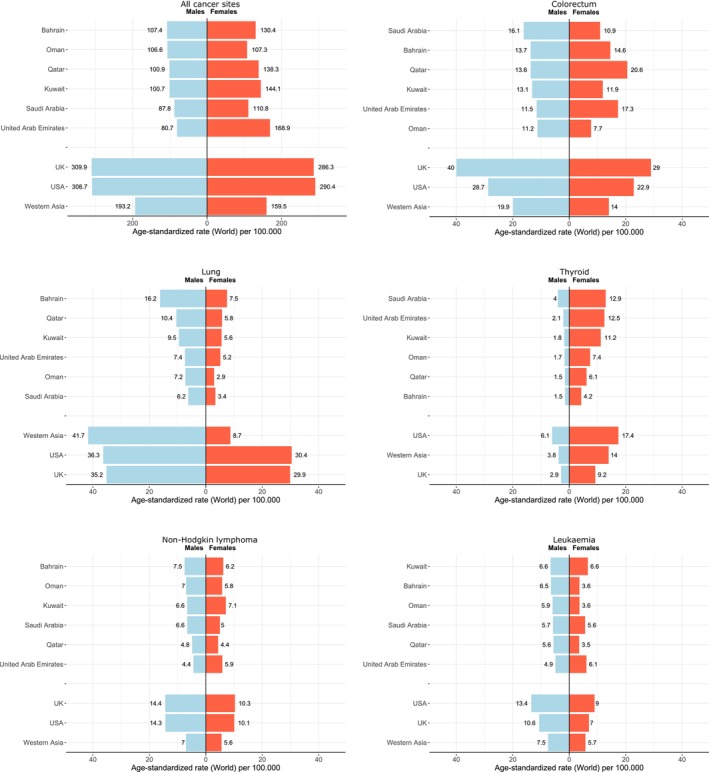
ASR incidence rates in males and females for all sites, colorectum, lung, thyroid, non‐Hodgkin lymphoma, and leukemia in the GCC countries compared to other countries and regions, GLOBOCAN 2020.

Overall, cancer incidence in the GCC countries was higher in females than males, with the female‐to‐male ratio ranging from unity in Oman to 2.0 in the UAE (Table 1; Figure 4). The overall ASRs for colorectal cancer incidence in the GCC countries combined, as well as in the US and the UK, were higher in males than in females, but this was not the case across all GCC countries (Figure 4, Table S2). Colorectal cancer incidence rates were higher in females than males in Qatar (20.6 vs. 13.6), UAE (17.3 vs. 11.5), and Bahrain (14.6 vs. 13.7), respectively. For thyroid cancer, incidence rates were higher in females than males across the GCC countries, with the overall ASR across GCC countries in females at 11.7 versus 3.0 among males, an observation seen in many of the individual GCC countries (Table S5).

### 3.4 Cancer incidence and mortality prediction by 2040

**FIGURE 5:**
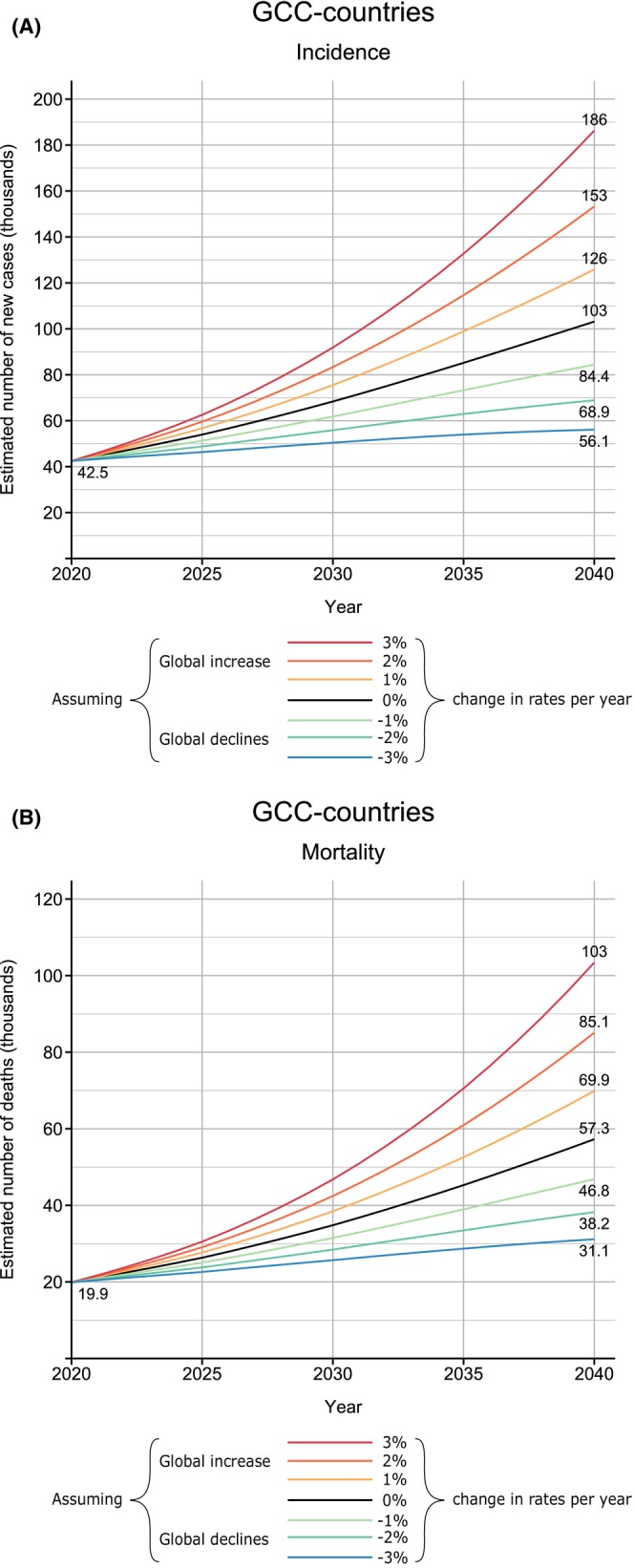
(A) Predicted number of new cases from all cancers in GCC countries assuming seven scenarios of annual change in global rates between 2020 and 2040, GLOBOCAN 2020. (B) Predicted number of cancer mortality from all cancers in GCC countries assuming seven scenarios of annual change in global rates between 2020 and 2040, GLOBOCAN 2020.

Despite the low rates in the region, projections for 2040 indicate there will be a rapid increase in the number of new cancer cases and cancer deaths over the next two decades, even if rates remain unchanged (Figure 5A,B). Under this scenario, around 103,000 cancer cases are expected to occur in the GCC countries in 2040, compared to 42,500 in 2020, while the cancer deaths are expected to reach over 57,300 compared to 19,900 deaths in 2020. Even with a 3% annual decrease in ASRs, the number of new cases and deaths in the GCC countries would still increase by 32% and 56% by 2040, respectively (Figure 5A,B). In terms of the cancer incidence burden, Qatar is estimated to have the largest increase in cancer cases, a 270% increase by 2040 compared to the current estimates in 2020, followed by UAE (230%), with the smallest predicted rise in Saudi Arabia still representing doubling of the incidence burden (116%) (Table 2). The GCC countries are also estimated to have a rapidly increasing cancer mortality burden by 2040, with Qatar estimated to have a 362% increase in the numbers of cancer deaths by 2040 relative to 2020, followed by UAE (330%), while the smallest increment in cancer deaths expected is in Saudi Arabia (152%), assuming rates remain fixed to those estimated in 2020 (Table 2; Figure 5A,B).

**TABLE 2:** Percentage changes in all cancer incidences and mortality in the GCC countries in 2040 compared to the baseline number in 2020, excluding non‐melanoma skin cancer.

Incidence (both sexes)				Mortality (both sexes)		
Country	Cases 2020	Cases 2040	% increase	Cases 2020	Cases 2040	% increase
Bahrain	1205	3239	168.8	592	1890	219.3
Kuwait	3824	10,684	179.4	1718	5963	247.1
Oman	3664	8360	128.2	2019	5281	161.6
Qatar	1472	5498	273.5	703	3249	362.2
Saudi Arabia	27,578	59,694	116.5	12,986	32,728	152
UAE	4732	15,667	231.1	1877	8154	334.4
All GCC countries	42,475	103,142	142.8	19,895	57,265	187.8

Abbreviations: GCC, Gulf Cooperation Council; UAE, United Arab Emirates.

## 4 DISCUSSION

To our knowledge, this is the first study to comprehensively compare national estimates of cancer incidence and mortality in the GCC countries, providing data‐driven evidence to guide cancer control policies in the GCC countries based on the most recent GLOBOCAN estimates for 2020 and projections up to the year 2040. Cancer incidence rates in the GCC countries tend to be one‐third of those observed in high‐income Western countries, with the overall GCC incidence rate <100 per 100,000, contrasting with the rates close to 300 in the US or the UK. Variations in mortality rates for all cancers combined were lesser (52.3 in GCC compared with 85.7 in the US and 99.6 in the UK), with a similar magnitude of breast and colorectal cancer mortality rates in some GCC countries. Colorectal and breast cancer were the leading cancers in the GCC countries in terms of incidence, with very low rates observed for specific cancer types, including cervix, lung, and alcohol‐related cancers such as liver and cancers of the upper digestive tract.^16^

Cancer is emerging as a major public health problem in the Gulf region, not least given the scenario that even a 3% annual decrease in overall cancer rates across GCC countries would still lead to 32% and 56% respective increases in the number of new cases and deaths by 2040. The exceptional rise in the cancer burden in the GCC region reflects improvements in health services and subsequently increased life expectancy alongside population aging and growth.^2^, ^5^, ^6^, ^17^ Additionally, the increasing prevalence of modifiable risk factors such as smoking, obesity, sedentary lifestyle, physical inactivity, and unhealthy diet has further driven cancer incidence increases in the GCC region.^18^ In particular, the prevalence of overweight and obesity has tripled in the GCC countries over the past four decades.^19^ Yet there are many positive efforts to prioritize cancer prevention that focus on well‐established cancer‐related risk factors, including the promotion of a healthy lifestyle through the taxing of tobacco and sweetened beverages and introduction of the human papillomavirus (HPV) vaccine (in Saudi Arabia and UAE).^6^, ^7^, ^17^ Due to strict alcohol regulations in the GCC countries, the region continues to have one of the lowest burdens of cancer attributable to alcohol consumption.^20^ Moreover, recent estimates of the proportion of cancer cases attributable to infections were relatively low but variable (ranging from 7% in Bahrain to 13% in Oman),^21^ while none of the main infection‐related cancer types (gastric, liver, and cervical) were among the five most common cancers in the region (other than in Oman, where gastric cancer is the third most common cancer in men and the fourth in both sexes).

Female breast cancer ranks first in the GCC countries in terms of incidence and in second for mortality. The low rates of breast cancer incidence in Saudi Arabia (28.8/100,000) at present relative to the other GCC countries might reflect variations in the intensity of screening efforts and the prevalence of risk factors in the past.^3^ Mainly, opportunistic national breast cancer screening programs are available in all GCC countries and have been covered by universal healthcare since 2007.^3^, ^6^ These programs have focused on improving awareness, training women in breast self‐examination, and providing mammography screening through designated centers and mobile units.^6^ The current screening guidelines encourage women to have mammograms starting at age 40 in all countries except Qatar, which starts at age 45, with a frequency ranging from 1 to 3 years.^6^ Yet, the uptake of breast cancer screening is still limited (varying by country from 5%–16% of eligible women)^22^ in most of the GCC countries, which results in a higher proportion of late‐stage diagnoses.^3^, ^6^ It is therefore important to continue to monitor breast cancer early detection programs in each country, evaluate their effectiveness and barriers to low uptake, and embed these activities within each country's national cancer control plan.

Colorectal cancer incidence ranked first in the region in terms of mortality and second in terms of incidence. The patterns and profile in the region likely reflect the increasing prevalence of unhealthy diets, lack of physical activity and sedentary lifestyles, high consumption of meat, and obesity, all of which are established risk factors for colorectal cancer.^17^, ^23^ With the number of new cases of colorectal cancer expected to increase by 150% by 2040, the GCC countries have already started several initiatives to lower the prevalence of established risk factors, including implementing the World Health Organization Framework Convention on Tobacco Control,^24^ promoting physical activity, and taxing sugary and sweetened drinks.^6^, ^7^, ^17^ In addition, colorectal cancer is emerging as a leading cause of cancer death in the GCC countries. This is a unique profile for cancer mortality in contrast to many countries both within the Western Asian region and worldwide, where it is commonplace that lung and prostate cancers are the predominant forms of cancer death. Although all GCC countries have early colorectal cancer detection programs targeting the ages 40–75—using mainly the fecal immunochemical test and colonoscopy—they are still in an early phase.^6^, ^25^ Research is needed in terms of an evaluation of the uptake, challenges, cost‐effectiveness, risk stratification, and overall benefits at the population level in terms of impact on colorectal cancer incidence and mortality rates.

A recent study estimated that the direct medical costs of colon and breast cancer in the GCC countries in 2019 were $158 million,^7^ emphasizing the need for future investment in national cancer control plans across the region to tackle the increasing cancer burden.^26^ Future research should focus on the barriers to breast and colorectal cancer early detection in the region and design and implement tailored and context‐specific cancer control policies. An additional focus for research should be stratified cancer screening and related cost‐effectiveness studies to improve the impact of cancer detection efforts.

Lung cancer in the GCC countries is not among the top five common cancers in both sexes combined, except in Bahrain, where it is the third most common cancer in both sexes combined. Lung cancer remains more common in males than in females, likely reflecting the lower prevalence of smoking among women in past decades in the GCC countries.^27^ Although smoking rates have been recently increasing in the GCC countries,^6^, ^27^ the proportion of all cancer cases among both sexes in 2018 attributable to tobacco smoking in all GCC countries is around 16%, lower than many countries in western Asia.^4^ As a result of the overall low smoking rates and variations between male and female rates, lung cancer is ranked as the third leading cause of cancer mortality in both sexes combined in the GCC region, second in males and fifth in females. While the GCC countries have made great progress aligning with the global efforts in tobacco control,^6^ it is important to tackle the recent trends of increasing uptake of smoking among young women in many of the GCC countries.^6^, ^27^

Thyroid cancer was the third most common cancer in the GCC countries in both sexes combined and the second most common cancer among females in all countries except Qatar and Bahrain. In the last two decades, thyroid cancer has increased substantially in the GCC countries, which in part reflects the effect of overdiagnosis, as seen in many countries,^28^ although an increase in exposure to risk factors such as the increase in the prevalence of overweight and obesity in the GCC countries cannot be discounted.^19^, ^29^ The ASR incidence is higher in females than males across the GCC countries, with the overall ASR for incidence in all GCC countries combined in females being 11.7 versus 3.0 for males, similar to the case in many countries.^28^ Although the incidence rates in GCC countries are higher than in the UK, the mortality rates are still low and similar to those in the US and the UK.

The increasing burden of cancer in the GCC countries requires better surveillance to assess and monitor progress in cancer control policies. Despite the development and advances in health information systems, the GCC states still face challenges in vital statistics data, particularly in accurate coding and recording the cause of death in cancer cases.^13^, ^30^ While the suboptimal quality of the cause of death data can lead to an underestimation of cancer mortality, an additional challenge for population‐based cancer registries is the incompleteness of follow‐up (vital status) information that is needed to estimate cancer survival.^30^ A challenge specific to the GCC countries is the collection of diagnostic information and follow‐up of non‐national (expatriate) populations due to their high mobility.^13^, ^31^ Further work is needed to overcome the present lack of robust mortality and survival in the GCC countries, in particular in improving information on the underlying cause of deaths registered on death certificates.^6^ In addition, it is vital to enable access and improve data linkages between the existing death registries and population‐based cancer registries, given that several countries have a unique identification number that enables such linkages.^6^, ^32^

This study has several limitations. The cancer mortality estimates are limited in the GCC countries, given the lack of vital registration data in the region.^6^, ^26^, ^32^ In addition, these estimates were developed using data collected prior to the COVID‐19 pandemic, which has been shown to affect the cancer registration process and care outcomes.^33^ Furthermore, to provide an overview of the cancer incidence rates in GCC countries compared to other countries, cancer incidence rates were compared to those in the US and the UK, both of which have well‐established cancer screening programs, which may contribute to the high cancer incidence rates compared to the GCC countries.^34^, ^35^ This study does, however, provide a timely review of the estimated cancer incidence and mortality patterns between the GCC countries and their underlying populations and documents the large increase in cancer incidence and mortality expected in the GCC countries over the coming decades. Such an exposition of current patterns, recent trends, and future projections provides evidence for local policymakers seeking to develop national cancer control interventions and serves as a baseline reference for further studies in the GCC region. Despite the existence of estimates, the GCC countries need to make a concerted effort to improve the generation of systematic and continuously recorded cancer data by strengthening and harmonizing existing population‐based cancer registries in the Gulf region.

## AUTHOR CONTRIBUTIONS

S alessy, SAA, AZ, and FB conceptualized and designed the study. JV accessed the data and did the formal analysis. S alessy, AZ, and FB wrote the report and interpreted the data. All authors critically reviewed the manuscript for important intellectual content and drafting of the manuscript. All authors had full access to all the data in the study and had final responsibility for the decision to submit for publication.

## FUNDING INFORMATION

No external funds were received.

## CONFLICT OF INTEREST STATEMENT

The authors declare no conflicts of interest.

## Supporting information


Data S1.

## Data Availability

The data used in this study are retrievable from the publicly available platform, the Global Cancer Observatory (GLOBOCAN, https://gco.iarc.fr/).
