# Supplementary material for: The current and future cancer burden in the Gulf Cooperation Council (GCC) countries
Source: Cancer Med. 2024 Sep 16;13(17):e70141. doi: 10.1002/cam4.70141 (PMC11403302; doi:10.1002/cam4.70141)
Supplement: Supplementary file 1 — Data S1. [file CAM4-13-e70141-s001.docx]

**Article Title**: The current and future cancer burden in the Gulf Cooperation Council (GCC) countries

Content:

[Supplementary Table 1: Breast cancer incidence and mortality in the Gulf Cooperation Council countries in GLOBOCAN 2020 2](#_Toc173060572)

[Supplementary Table 2: Colorectal cancer incidence and mortality in the Gulf Cooperation Council countries in GLOBOCAN 2020 3](#_Toc173060573)

[Supplementary Table 3: Lung cancer incidence and mortality in the Gulf Cooperation Council countries in GLOBOCAN 2020 4](#_Toc173060574)

[Supplementary Table 4: Prostate cancer incidence and mortality in the Gulf Cooperation Council countries in GLOBOCAN 2020 5](#_Toc173060575)

[Supplementary Table 5: Thyroid cancer incidence and mortality in the Gulf Cooperation Council countries in GLOBOCAN 2020 6](#_Toc173060576)

[Supplementary Table 6: Non-Hodgkin’s lymphoma cancer incidence and mortality in the Gulf Cooperation Council countries in GLOBOCAN 2020 7](#_Toc173060577)

[Supplementary Table 7: Leukemia cancer incidence and mortality in the Gulf Cooperation Council countries in GLOBOCAN 2020 8](#_Toc173060578)

[Supplementary Table 8: Liver cancer incidence and mortality in the Gulf Cooperation Council countries in GLOBOCAN 2020 9](#_Toc173060579)

Supplementary Table 1: Breast cancer incidence and mortality in the Gulf Cooperation Council countries in GLOBOCAN 2020

|  | **INCIDENCE** | | | | | | | **MORTALITY** | | | | | |
| --- | --- | --- | --- | --- | --- | --- | --- | --- | --- | --- | --- | --- | --- |
|  | **Total** | | | **Male** | | **Female** | | **Total** | | **Male** | | **Female** | |
| **Country** | **N** | **(%)** | | **N** | **ASR** | **N** | **ASR** | **N** | **(%)** | **N** | **ASR** | **N** | **ASR** |
| Bahrain | 244 | | 3.6 | - | - | 244 | 44.1 | 66 | 3.6 | - | - | 66 | 13.6 |
| Kuwait | 791 | | 11.6 | - | - | 791 | 50.3 | 224 | 12 | - | - | 224 | 17.0 |
| Oman | 558 | | 8.2 | - | - | 558 | 38.5 | 195 | 10.5 | - | - | 195 | 14.5 |
| Qatar | 218 | | 3.2 | - | - | 218 | 42.8 | 57 | 3.1 | - | - | 57 | 13.2 |
| Saudi Arabia | 3954 | | 58.2 | - | - | 3954 | 28.8 | 1095 | 58.9 | - | - | 1095 | 8.9 |
| UAE | 1030 | | 15.2 | - | - | 1030 | 58.5 | 222 | 11.9 | - | - | 222 | 16.6 |
| All GCC countries | 6795 | | 100 | - | - | 6795 | 34.4 | 1859 | 100 | - | - | 1859 | 10.6 |

Supplementary Table 2: Colorectal cancer incidence and mortality in the Gulf Cooperation Council countries in GLOBOCAN 2020

|  | **INCIDENCE** | | | | | | **MORTALITY** | | | | | |
| --- | --- | --- | --- | --- | --- | --- | --- | --- | --- | --- | --- | --- |
|  | **Total** | | **Male** | | **Female** | | **Total** | | **Male** | | **Female** | |
| **Country** | N | (%) | N | ASR | N | ASR | N | (%) | N | ASR | N | ASR |
| Bahrain | 147 | 2.6 | 83 | 13.7 | 64 | 14.6 | 65 | 2.4 | 36 | 6.8 | 29 | 7.5 |
| Kuwait | 411 | 7.3 | 256 | 13.1 | 155 | 11.9 | 188 | 6.8 | 118 | 7.0 | 70 | 6.1 |
| Oman | 385 | 6.8 | 289 | 11.2 | 96 | 7.8 | 213 | 7.7 | 161 | 6.2 | 52 | 4.5 |
| Qatar | 174 | 3.1 | 112 | 13.7 | 62 | 20.6 | 80 | 2.9 | 51 | 8.0 | 29 | 10.9 |
| Saudi Arabia | 4007 | 71.1 | 2756 | 16.1 | 1251 | 10.9 | 1996 | 72.3 | 1374 | 8.7 | 622 | 5.6 |
| UAE | 510 | 9.1 | 313 | 11.5 | 197 | 17.3 | 218 | 7.9 | 136 | 6.2 | 82 | 8.7 |
| All GCC countries | 5634 | 100 | 3809 | 14.4 | 1825 | 11.3 | 2760 | 100 | 1876 | 7.7 | 884 | 5.7 |

Supplementary Table 3: Lung cancer incidence and mortality in the Gulf Cooperation Council countries in GLOBOCAN 2020

|  | **INCIDENCE** | | | | | | **MORTALITY** | | | | | |
| --- | --- | --- | --- | --- | --- | --- | --- | --- | --- | --- | --- | --- |
|  | **Total** | | **Male** | | **Female** | | **Total** | | **Male** | | **Female** | |
| **Country** | **N** | **(%)** | **N** | **ASR** | **N** | **ASR** | **N** | **(%)** | **N** | **ASR** | **N** | **ASR** |
| Bahrain | 96 | 5.1 | 65 | 16.2 | 31 | 7.5 | 84 | 5.1 | 59 | 15.1 | 25 | 6.1 |
| Kuwait | 190 | 10 | 142 | 9.5 | 48 | 5.6 | 164 | 10 | 124 | 8.7 | 40 | 4.8 |
| Oman | 137 | 7.2 | 108 | 7.2 | 29 | 3 | 127 | 7.7 | 101 | 6.7 | 26 | 2.7 |
| Qatar | 87 | 4.6 | 71 | 10.4 | 16 | 5.8 | 77 | 4.7 | 63 | 9.6 | 14 | 5.5 |
| Saudi Arabia | 1157 | 61.1 | 852 | 6.2 | 305 | 3.4 | 1001 | 61 | 719 | 5.5 | 282 | 3.2 |
| UAE | 226 | 11.9 | 169 | 7.4 | 57 | 5.2 | 187 | 11.4 | 147 | 6.6 | 40 | 4 |
| All GCC countries | 1893 | 100 | 1407 | 7 | 486 | 3.8 | 1640 | 100 | 1213 | 6.2 | 427 | 3.4 |

Supplementary Table 4: Prostate cancer incidence and mortality in the Gulf Cooperation Council countries in GLOBOCAN 2020

|  | **INCIDENCE** | | | | | | **MORTALITY** | | | | | |
| --- | --- | --- | --- | --- | --- | --- | --- | --- | --- | --- | --- | --- |
|  | **Total** | | **Male** | | **Female** | | **Total** | | **Male** | | **Female** | |
| **Country** | **N** | **(%)** | **N** | **ASR** | **N** | **ASR** | **N** | **(%)** | **N** | **ASR** | **N** | **ASR** |
| Bahrain | 51 | 3.3 | 51 | 13.3 | - | - | 12 | 3 | 12 | 4.5 | - | - |
| Kuwait | 255 | 16.3 | 255 | 19.6 | - | - | 52 | 12.8 | 52 | 6.6 | - | - |
| Oman | 189 | 12.1 | 189 | 13.8 | - | - | 73 | 18 | 73 | 6.0 | - | - |
| Qatar | 104 | 6.7 | 104 | 21.1 | - | - | 18 | 4.4 | 18 | 4.8 | - | - |
| Saudi Arabia | 693 | 44.4 | 693 | 7.0 | - | - | 204 | 50.2 | 204 | 2.5 | - | - |
| UAE | 270 | 17.3 | 270 | 13.4 | - | - | 47 | 11.6 | 47 | 3.4 | - | - |
| All GCC countries | 1562 | 100 | 1562 | 9.8 | - | - | 406 | 100 | 406 | 3.2 | - | - |

Supplementary Table 5: Thyroid cancer incidence and mortality in the Gulf Cooperation Council countries in GLOBOCAN 2020

|  | **INCIDENCE** | | | | | | **MORTALITY** | | | | | |
| --- | --- | --- | --- | --- | --- | --- | --- | --- | --- | --- | --- | --- |
|  | **Total** | | **Male** | | **Female** | | **Total** | | **Male** | | **Female** | |
| **Country** | **N** | **(%)** | **N** | **ASR** | **N** | **ASR** | **N** | **(%)** | **N** | **ASR** | **N** | **ASR** |
| Bahrain | 36 | 0.9 | 10 | 1.5 | 26 | 4.2 | 3 | 0.8 | 2 | 0.6 | 1 | 0.2 |
| Kuwait | 264 | 6.9 | 54 | 1.8 | 210 | 11.2 | 25 | 7.1 | 6 | 0.4 | 19 | 1.7 |
| Oman | 185 | 4.9 | 50 | 1.6 | 135 | 7.4 | 21 | 5.9 | 7 | 0.4 | 14 | 1.2 |
| Qatar | 77 | 2 | 35 | 1.5 | 42 | 6.1 | 8 | 2.3 | 4 | 0.3 | 4 | 1.6 |
| Saudi Arabia | 2833 | 74.6 | 884 | 4.0 | 1949 | 12.9 | 263 | 74.5 | 108 | 0.8 | 155 | 1.5 |
| UAE | 405 | 10.7 | 93 | 2.1 | 312 | 12.4 | 33 | 9.3 | 10 | 0.7 | 23 | 2.6 |
| All GCC countries | 3800 | 100 | 1126 | 3.0 | 2674 | 11.7 | 353 | 100 | 137 | 0.7 | 216 | 1.5 |

Supplementary Table 6: Non-Hodgkin’s lymphoma cancer incidence and mortality in the Gulf Cooperation Council countries in GLOBOCAN 2020

|  | **INCIDENCE** | | | | | | **MORTALITY** | | | | | |
| --- | --- | --- | --- | --- | --- | --- | --- | --- | --- | --- | --- | --- |
|  | **Total** | | **Male** | | **Female** | | **Total** | | **Male** | | **Female** | |
| **Country** | **N** | **(%)** | **N** | **ASR** | **N** | **ASR** | **N** | **(%)** | **N** | **ASR** | **N** | **ASR** |
| Bahrain | 71 | 2.8 | 45 | 7.5 | 26 | 6.2 | 24 | 2 | 15 | 3.1 | 9 | 2.4 |
| Kuwait | 230 | 9.1 | 136 | 6.6 | 94 | 7.1 | 83 | 7.1 | 49 | 3.0 | 34 | 3.5 |
| Oman | 241 | 9.6 | 172 | 7.0 | 69 | 5.8 | 130 | 11.1 | 91 | 4.7 | 39 | 3.5 |
| Qatar | 88 | 3.5 | 68 | 4.8 | 20 | 4.4 | 38 | 3.2 | 31 | 2.7 | 7 | 2.1 |
| Saudi Arabia | 1698 | 67.5 | 1141 | 6.6 | 557 | 5.0 | 827 | 70.4 | 555 | 3.9 | 272 | 2.7 |
| UAE | 189 | 7.5 | 115 | 4.4 | 74 | 5.9 | 73 | 6.2 | 46 | 2.8 | 27 | 3.2 |
| All GCC countries | 2517 | 100 | 1677 | 6.1 | 840 | 5.2 | 1175 | 100 | 787 | 3.6 | 388 | 2.7 |

Supplementary Table 7: Leukemia cancer incidence and mortality in the Gulf Cooperation Council countries in GLOBOCAN 2020

|  | **INCIDENCE** | | | | | | **MORTALITY** | | | | | |
| --- | --- | --- | --- | --- | --- | --- | --- | --- | --- | --- | --- | --- |
|  | **Total** | | **Male** | | **Female** | | **Total** | | **Male** | | **Female** | |
| **Country** | **N** | **(%)** | **N** | **ASR** | **N** | **ASR** | **N** | **(%)** | **N** | **ASR** | **N** | **ASR** |
| Bahrain | 56 | 2.2 | 38 | 6.5 | 18 | 3.6 | 34 | 2.2 | 21 | 3.2 | 13 | 2.7 |
| Kuwait | 203 | 8.1 | 124 | 6.6 | 79 | 6.6 | 120 | 7.8 | 71 | 3.9 | 49 | 4.2 |
| Oman | 212 | 8.4 | 155 | 5.9 | 57 | 3.6 | 156 | 10.1 | 115 | 4.4 | 41 | 2.8 |
| Qatar | 93 | 3.7 | 73 | 5.6 | 20 | 3.5 | 60 | 3.9 | 47 | 3.7 | 13 | 2.4 |
| Saudi Arabia | 1676 | 66.7 | 958 | 5.8 | 718 | 5.6 | 1032 | 66.8 | 593 | 3.5 | 439 | 3.4 |
| UAE | 272 | 10.8 | 164 | 4.9 | 108 | 6.1 | 142 | 9.2 | 86 | 2.3 | 56 | 3.4 |
| All GCC countries | 2512 | 100 | 1512 | 5.6 | 1000 | 5.4 | 1544 | 100 | 933 | 3.3 | 611 | 3.2 |

Supplementary Table 8: Liver cancer incidence and mortality in the Gulf Cooperation Council countries in GLOBOCAN 2020

|  | **INCIDENCE** | | | | | | **MORTALITY** | | | | | |
| --- | --- | --- | --- | --- | --- | --- | --- | --- | --- | --- | --- | --- |
|  | **Total** | | **Male** | | **Female** | | **Total** | | **Male** | | **Female** | |
| **Country** | **N** | **(%)** | **N** | **ASR** | **N** | **ASR** | **N** | **(%)** | **N** | **ASR** | **N** | **ASR** |
| Bahrain | 36 | 2.3 | 22 | 4.0 | 14 | 3.4 | 34 | 2.2 | 21 | 4.0 | 13 | 3.3 |
| Kuwait | 128 | 8.1 | 93 | 5.4 | 35 | 4.1 | 122 | 8.1 | 89 | 5.1 | 33 | 4.0 |
| Oman | 128 | 8.1 | 100 | 5.8 | 28 | 2.5 | 121 | 8 | 95 | 5.4 | 26 | 2.4 |
| Qatar | 56 | 3.6 | 45 | 5.7 | 11 | 4 | 54 | 3.6 | 44 | 5.7 | 10 | 3.9 |
| Saudi Arabia | 1145 | 72.7 | 841 | 6.8 | 304 | 3.3 | 1105 | 73.1 | 819 | 6.6 | 286 | 3.1 |
| UAE | 83 | 5.3 | 53 | 2.8 | 30 | 3.2 | 76 | 5 | 48 | 2.6 | 28 | 3.2 |
| All GCC countries | 1576 | 100 | 1154 | 6 | 422 | 3.2 | 1512 | 100 | 1116 | 5.8 | 396 | 3.1 |
